# Medicare/Medicaid Insurance Status Is Associated With Reduced Lower Bilateral Knee Arthroplasty Utilization and Higher Complication Rates

**DOI:** 10.5435/JAAOSGlobal-D-21-00016

**Published:** 2022-04-26

**Authors:** Bella Mehta, Kaylee Ho, Jennifer Bido, Stavros G. Memtsoudis, Michael L. Parks, Linda Russell, Susan M. Goodman, Said Ibrahim

**Affiliations:** From the Department of Medicine, Hospital for Special Surgery, New York, NY (Dr. Mehta, Dr. Russell, and Dr. Goodman); the Department of Medicine (Dr. Mehta, Dr. Memtsoudis, Dr. Parks, Dr. Russell, and Dr. Goodman), and the Department of Population Health Sciences (Dr. Ho), Weill Cornell Medicine, New York, NY; the Department of Orthopedics (Dr. Bido and Dr. Parks), and the Department of Anesthesiology (Dr. Memtsoudis), Hospital for Special Surgery, New York, NY; and the Department of Healthcare Policy & Research, Weill Cornell Medicine, New York, NY (Dr. Ibrahim).

## Abstract

Whether to undergo bilateral total knee arthroplasty (BTKA) depends on patient and surgeon preferences. We used the National Inpatient Sample to compare temporal trends in BTKA utilization and in-hospital complication rates among TKA patients ≥50 with Medicare/Medicaid versus private insurance from 2007 to 2016. We used multivariable logistic regression to assess the association between insurance type and trends in utilization and complication rates adjusting for individual-, hospital-, and community-level covariates, using unilateral TKA (UTKA) for reference. Discharge weights were used for nationwide estimates. About 132,400 (49.5%) Medicare/Medicaid patients and 135,046 (50.5%) privately insured patients underwent BTKA. Among UTKA patients, 62.7% had Medicare/Medicaid, and 37.3% had private insurance. Over the study period, BTKA utilization rate decreased from 7.18% to 5.63% among privately insured patients and from 4.59% to 3.13% among Medicaid/Medicare patients (*P* trend difference <0.0001). In multivariable analysis, Medicare/Medicaid patients were less likely to receive BTKA than privately insured patients. Although Medicare/Medicaid patients were more likely to develop in-hospital complications after UTKA (adjusted odds ratio, 1.06; 95% confidence interval, 1.002 to 1.12; *P* = 0.04), this relationship was not statistically significant for BTKAs. In this nationwide sample of TKA patients, BTKA utilization rate was higher in privately insured patients compared with Medicare/Medicaid patients. Furthermore, privately insured patients had lower in-hospital complication rates than Medicare/Medicaid patients.

Total knee arthroplasty (TKA) is one of the most common surgeries performed in the United States, with >600,000 procedures occurring annually.^1^ As the proportion of the US population over the age of 65 years increases, the use of TKA is projected to increase to >1.2 million cases annually by 2030.^1^ Medicare is the primary payer for >60% of TKA and total hip arthroplasty (THA).^1,2^ As such, given the projected increases in cases, Centers for Medicare & Medicaid Services (CMS) spending on total joint arthroplasty is expected to increase to $50 billion annually.^1,2^ Policies aiming to decrease Medicare/Medicaid spending have focused on total joint arthroplasty, as it is the largest expenditure paid by CMS.^3^

One option that has been suggested to decrease healthcare costs is the use of simultaneous bilateral TKA (BTKA). Proponents of BTKA tout its use of a single anesthetic, shorter overall surgical time, less time lost from work, and lower overall use of narcotics.^4-6^ However, there are questions about its safety. Some studies report no increase in complications in unilateral TKA (UTKA) versus BTKA,^6-8^ whereas others report increased risk of postoperative venous thromboembolism and cardiorespiratory complications.^9-11^ Ultimately, the choice in treatment strategy continues to be debated and largely depends on patient and surgeon preferences, as guidelines vary per institution.^9^

Prior literature has framed BTKA as a cost-effective procedure.^12,13^ However, this literature, which focuses on national healthcare spending, does not consider the financial incentives for individual practitioners and institutions. Analysis of CMS reimbursement trends for total joint arthroplasty demonstrates that surgeon reimbursement has decreased by >30% over the past 20 years.^3^ Furthermore, in 1992, CMS decreased reimbursement for a second total joint arthroplasty by 50% if performed within 90 days of a first total joint arthroplasty.^14^ An initial assessment by Della Valle et al into the effect of CMS reimbursement changes on the number of BTKA performed found no difference in BTKA rates during the initial years after the policy change. However, this analysis was limited to the year 1996 and therefore did not include the more recent period of decline in Medicare/Medicaid reimbursements. In addition, this analysis did not compare the Medicare population with those with private insurance. Notably, private insurance quickly followed suit and also decreased the reimbursement for a second total joint arthroplasty by 50%. Therefore, it is not clear whether insurance status influences BTKA utilization rate.

The primary objective of this analysis was to examine whether variations in utilization of BTKA over time are associated with insurance type. Given the decrease in overall CMS reimbursement and possible physician incentives to minimize financial loss, we hypothesized that BTKA utilization over time would be less in the Medicare/Medicaid population relative to populations with private insurances that offer higher reimbursements. Our next objective was to examine the relationship between in-hospital complication rates of BTKA and insurance type. We hypothesized that once controlling for patient demographics, there would be no difference in complications rates after BTKA based on insurance status. Finally, we aimed to investigate whether race, which has been independently associated with BTKA utilization,^15^ modifies the relationship between insurance status and BTKA utilization. We hypothesized that once controlling for race, insurance status would be independently associated with BTKA utilization over time.

## Methods

### Data Source

We analyzed data from the National Inpatient Sample (NIS) from 2007 through 2016. NIS is the largest publicly available inpatient database in the United States. It is sponsored by the Agency for Healthcare Research and Quality and the Healthcare Cost and Utilization Project. Unweighted, it contains data from >7 million hospital stays each year. Weighted, it provides estimates on more than 35 million hospitalizations nationally. Before 2012, the NIS included all discharge data from more than 1,000 hospitals each year, approximating a 20% stratified sample of US community hospitals. NIS was redesigned in 2012, which now creates a sample of discharge records from all Healthcare Cost and Utilization Project–participating hospitals rather than all discharge records from a sample of hospitals. NIS represents >95% of the US population. Inpatient stay records in NIS include clinical and resource use information available from discharge abstracts derived from state-mandated hospital discharge reports. No unique patient identifiers are contained in the NIS for the hospitalization records. This study was deemed IRB exempt by Hospital for Special Surgery.

### Analytic Sample

**Figure 1:**
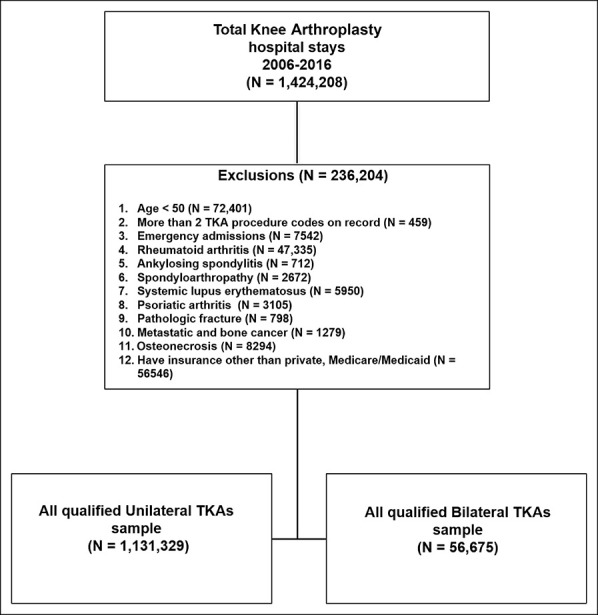
Healthcare Cost and Utilization Project National Inpatient Sample flowchart. TKA = total knee arthroplasty.

We obtained data on UTKA and BTKA hospitalizations with information on the primary insurance of interest (Medicare/Medicaid or Private). We used *International Classification of Diseases Ninth Revision* (*ICD-9*) codes from 2007 through September 2015 and *ICD-10* codes from October 2015 through December 2016 to identify patient diagnoses and procedures. We identified patients who underwent elective primary TKA using the *ICD-9* procedure code 81.54 for TKA and BTKA (when the procedure code was used twice in the same admission) from January 1, 2007, through September 30, 2015, and *ICD-10* procedure codes 0SRC0x and 0SRD0x thereafter.^16^ To narrow our sample to patients with osteoarthritis who had elective TKA, we excluded patients aged <50 years (because a large proportion of patients with end-stage osteoarthritis have surgery after age 50 years) and those with inflammatory arthritis (rheumatoid arthritis, ankylosing spondylitis, spondyloarthropathy, systemic lupus erythematosus, and psoriatic arthritis), emergency admissions, pathologic fracture, metastatic and/or bone cancer, and osteonecrosis (Figure 1). We also excluded those who had >2 knee arthroplasties on record during the index admission (likely administrative data set error). For study variables, clinical comorbidities were determined using discharge diagnosis codes, and an Elixhauser index was calculated.^17,18^ Patients with morbid obesity (not included in the Elixhauser index) were identified using *ICD-9* code 27801 and *ICD-10* code E660.

### Exposure Variables

The exposure variable of interest was primary insurance status, dichotomized into Medicare/Medicare versus private insurance. In addition, because race is known to be associated with disparities in joint arthroplasty utilization and outcomes, we included an interaction term for race (African American [AA] versus White) to see whether it is an effect modifier in the relationship.

### Key Study Outcomes

Our primary outcomes of interest were utilization rates and in-hospital complications over time. We assessed temporal trends in utilization of BTKA compared with UTKA between Medicare/Medicaid versus private insurance from 2007 to 2016. We also examined major in-hospital complication rates, such as postoperative myocardial infarction, prosthetic device complication, surgical wound infection, and venous thromboembolism.^19^ These were identified using *ICD-9/10* codes (Appendix Table 1, http://links.lww.com/JG9/A201). We studied differences in temporal trends in utilization of BTKA compared with UTKA between Medicare/Medicaid versus private insurance.

### Study Covariates

Study covariates included patient demographic characteristics such as age, sex, and race; ecological median household income per ZIP code; and hospital characteristics such as region, bed size, location, and teaching status. Patient clinical characteristics such as the Elixhauser comorbidity index and morbid obesity were also included as study covariates.

### Statistical Analysis

All categorical variables are reported as weighted frequencies and percentages using sampling weights and hospital clusters. The means and standard errors are reported for continuous variables. Community-level income is based on the median household income of the patient's ZIP code of residence. We examined the differences between baseline study characteristics stratified by insurance status using the Rao-Scott chi-square test (design-adjusted version of the Pearson chi-square test) for categorical variables and the two-sample independent *t*-test for continuous variables. The association between insurance and receipt of surgery, adjusted for patient age, sex, race, community median household income, Elixhauser comorbidity index and hospital volume, bed size, hospital region, and teaching status was analyzed using multivariable logistic regression models. To assess differences over time in BTKA utilization rate compared with UTKA by insurance type, we used an interaction term of insurance and time period.

Next, we conducted a stratified analysis for procedure type (BTKA and UTKA) to assess whether there is a trend difference in the relationship between insurance type and in-hospital complications. We adjusted for the same covariates as described above. In addition, we performed a model with an interaction term for race (White versus AA) and insurance type, adjusting for time and other covariates to examine whether race affects utilization differently in patients with Medicaid/Medicare versus private insurance. Unadjusted and adjusted odds ratios (aORs) of in-hospital complications were estimated. Multivariable models were adjusted for the same individual, ecological, and hospital-level variables described above.

All analyses accounted for the complex survey design, stratification, and clustering of data per NIS recommendations. All statistical tests are two sided, with statistical significance evaluated at the 0.05 alpha level. All analyses were conducted in SAS version 9.4 (SAS Institute) and R studio version 1.2.1335 and R version 3.6.0.

## Results

### Baseline Sample Characteristics

From 2007 through 2016, there were about 267,446 BTKAs (unweighted observations 56,675) and 5,322,105 UTKAs (unweighted observations 1,131,329) (Table 1). Overall, only 4.8% of patients underwent BTKA. Compared with privately insured patients, the mean age for Medicare/Medicaid insured patients is higher for both UTKA (mean = 71.7, SE = 0.03 versus mean = 60.7, SE = 0.04) and BTKA (mean = 70.6, SE = 0.07 versus mean = 59.7, SE = 0.05). There were a higher proportion of women in Medicaid/Medicare insured patients for both UTKA (64.6% versus 59.0%) and BTKA (58.1% versus 54.1%). There were a higher percentage of White patients undergoing BTKAs compared with UTKAs (87.3% in Medicare/Medicaid and 87.7% in private insurance undergoing BTKAs versus 83.4% in Medicare/Medicaid and 83.9% in private insurance undergoing UTKA).

**Table 1:** Baseline Characteristics and Outcomes by Total Knee Arthroplasty Type and Insurance, 2007 to 2016

Variable	Unilateral^a^			Bilateral^b^		
	Medicare/Medicaid N = 3,334,412 (62.7%)	Private N = 1,987,693 (37.3%)	*P* Value^c^	Medicare/Medicaid N = 132,400 (49.5%)	Private N = 135,046 (50.5%)	*P* Value^c^
Patient characteristics						
Age, mean (SE)	71.7 (0.03)	60.7 (0.04)	***	70.6 (0.07)	59.7 (0.05)	***
Sex, female, n (%)	2,151,721 (64.57)	1,172,586 (59.03)	***	76,931 (58.12)	73,061 (54.12)	***
Race, n (%)			***			
Black	203,455 (6.88)	128,893 (7.46)		6,816 (5.82)	6,806 (5.74)	
White	2,466,140 (83.36)	1,448,700 (83.87)		102,350 (87.32)	104,024 (87.74)	
Other	288,797 (9.76)	149,769 (8.67)		8041 (6.86)	7731 (6.52)	
Median household income (by patient's ZIP code), n (%)			***			***
0-25th percentile	777,239 (23.70)	369,236 (18.88)		29,240 (22.48)	23,433 (17.62)	
26th-50th percentile (median)	899,829 (27.44)	503,248 (25.73)		34,714 (26.69)	33,542 (25.23)	
51st-75th percentile	852,728 (26.01)	545,996 (27.91)		33,251 (25.57)	37,260 (28.02)	
76th-100th percentile	749,252 (22.85)	537,456 (27.48)		32,845 (25.26)	38,727 (29.13)	
Morbid obesity, n (%)	200,461 (6.01)	185,279 (9.32)	***	7,517 (5.68)	12,188 (9.02)	***
Elixhauser index,^d^ n (%)			***			***
0	345,397 (10.36)	336,632 (16.94)		16,023 (12.10)	24,038 (17.80)	
1-4	2,746,567 (82.37)	1,575,281 (79.25)		107,583 (81.26)	106,141 (78.60)	
≥5	242,448 (7.27)	75,779 (3.81)		8,794 (6.64)	4,867 (3.60)	
Hospital characteristics						
Region, n (%)			***			***
Northeast	548,916 (16.46)	331,976 (16.70)		31,365 (23.69)	33,337 (24.69)	
Midwest	894,031 (26.81)	575,407 (28.95)		35,438 (26.77)	39,676 (29.38)	
South	1,235,751 (37.06)	701,138 (35.27)		46,556 (35.16)	43,384 (32.13)	
West	655,714 (19.67)	379,171 (19.08)		19,040 (14.38)	18,650 (13.81)	
Bedsize, n (%)			***			***
Small	696,868 (20.96)	455,460 (22.98)		24,054 (18.24)	28,797 (21.42)	
Medium	895,491 (26.94)	530,732 (26.77)		36,632 (27.78)	36,715 (27.31)	
Large	1,731,687 (52.10)	996,037 (50.25)		71,159 (53.97)	68,947 (51.28)	
Volume (cases per year), n (%)			***			*
<100	1,276,836 (38.29)	681,665 (34.29)		42,390 (32.02)	40,868 (30.26)	
100-200	764,637 (22.93)	457,631 (23.02)		28,973 (21.88)	29,930 (22.16)	
>200	1,292,939 (38.78)	848,396 (42.68)		61,036 (46.10)	64,247 (47.57)	
Location/teaching status, n (%)			***			***
Rural	40,8253 (12.28)	194,572 (9.82)		20,238 (15.35)	16,446 (12.23)	
Urban nonteaching	1,401,475 (42.16)	819,832 (41.36)		48,175 (36.54)	49,990 (37.18)	
Urban teaching	1,514,318 (45.56)	967,825 (48.83)		63,431 (48.11)	68,023 (50.59)	
Outcomes						
Length of stay, mean (SE)	3.13 (0.01)	2.84 (0.01)	***	3.81 (0.03)	3.68 (0.04)	***
In-hospital complications	38,068 (1.14)	19,635 (0.99)	***	1,600 (1.21)	1,151 (0.85)	***
Acute myocardial infarction	7,290 (0.22)	1,585 (0.08)	***	558 (0.42)	208 (0.15)	***
Venous thromboembolism	2,920 (0.43)	1,616 (0.39)	*	138 (0.51)	125 (0.45)	
Device complications	10,381 (0.31)	6,700 (0.34)	*	40 (0.15)	37 (0.13)	
Wound complications	6,897 (0.21)	3,767 (0.19)		42 (0.15)	34 (0.12)	

All values were estimated using sampling weights and hospital clusters.

a Unilateral unweighted frequencies: N = 681,965 for Medicaid/Medicare; N = 407,172 for Private.

b Bilateral unweighted frequencies: N = 27,192 for Medicaid/Medicare; N = 27,687 for Private.

c *P* values are calculated based on the Rao-Scott chi-square test, a design-adjusted version of the Pearson chi-square test. Significance levels: *P < 0.05, **P < 0.01, ***P < 0.001.

d Clinical comorbidities were identified based on coding algorithms developed by Quan et al (enhanced Elixhauser version), using either *International Classification of Diseases Ninth Revision, Clinical Modulation*, or *International Classification of Diseases Tenth Revision, Clinical Modulation*, codes, as appropriate. The Elixhauser comorbidity index score is calculated based on the cumulative number of comorbidity conditions.

A higher proportion of AAs with private insurance (7.5%) compared with Medicare/Medicaid (6.9%) underwent UTKAs, although AAs were more likely to be Medicare/Medicaid (60.7%) insured. Patients with private insurance were more likely to have higher income, fewer comorbidities (Elixhauser index ≥5, 3.8% in private versus 7.3% in Medicare/Medicaid undergoing UTKAs; 3.6% in private versus 6.6% in Medicare/Medicaid undergoing BTKAs), and were admitted to high-volume or urban teaching hospitals more than those with Medicare/Medicaid insurance in both surgery groups. There were a higher proportion of patients with morbid obesity in private insurance (9.3% in UTKAs; 9% in BTKAs) compared with Medicare/Medicaid (6.0% in UTKAs; 5.7% in BTKAs).

### Relationship of Type of Insurance With Bilateral Total Knee Arthroplasty Utilization Over Time

**Figure 2:**
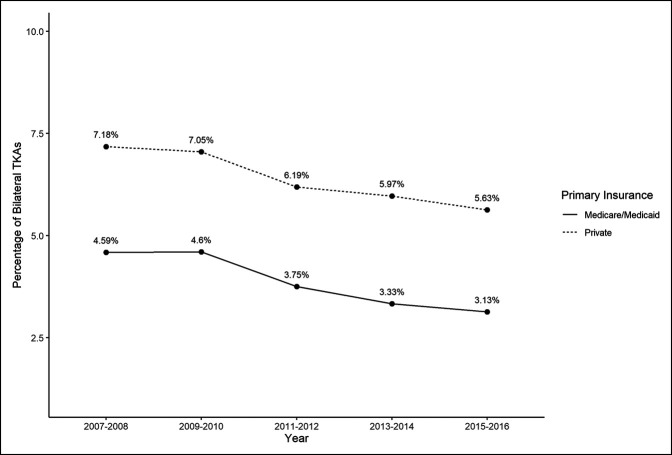
Graph showing the percentage of bilateral total knee arthroplasties (TKAs) among all TKAs by primary insurance.

Over the 10-year study period, the proportion of BTKA among all TKAs declined in both insurance groups, with overall lower utilization of BTKA from 7.18% in 2007 to 2008 to 5.63% in 2015 to 2016 in private insurance and from 4.6% in 2007 to 2008 to 3.1% in 2015 to 2016 in Medicare/Medicaid (Figure 2). The difference between utilization trends in the Medicare/Medicaid group and the private insurance group was statistically significant (*P* < 0.0001), suggesting that insurance type might influence utilization trends.

### In-Hospital Complication Rates by Insurance Type

**Figure 3:**
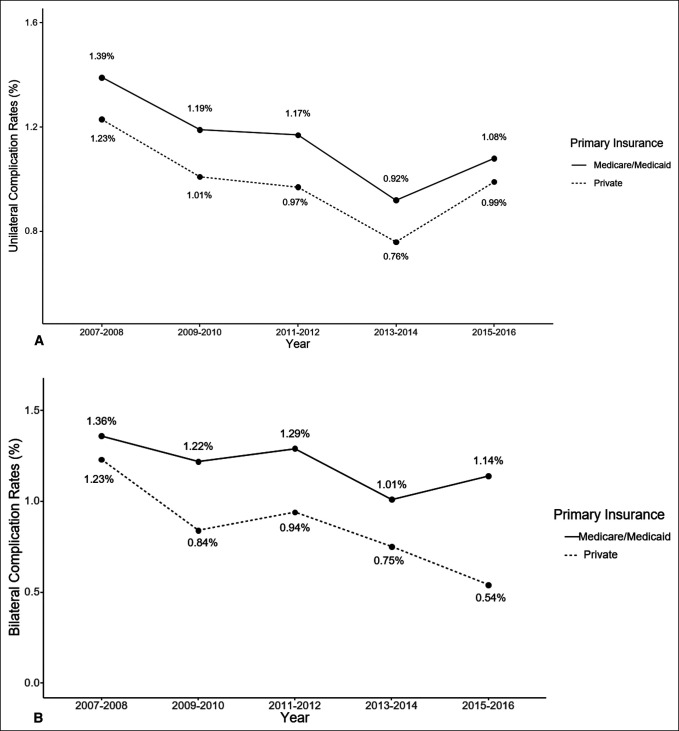
**A**, Graph showing unilateral total knee arthroplasty complication rates by primary insurance. **B**, Graph showing bilateral total knee arthroplasty complication rates by primary insurance.

**Figure 4:**
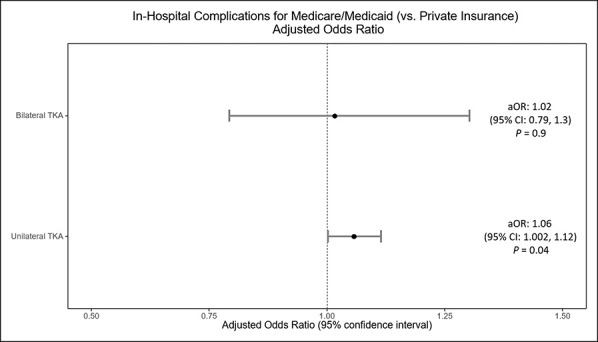
Graph showing adjusted models demonstrating the relationship of insurance and in-hospital complication rates. Models adjusted for individual demographics (age, race, and primary payer), ecological characteristics (median household income per ZIP code) and hospital characteristics (region, bed size, and location/teaching status). aOR = adjusted odds ratio, CI = confidence interval, TKA = total knee arthroplasty.

Among UTKAs, the overall in-hospital complication rate was 1.14% in Medicare/Medicaid patients compared with 0.99% in private insurance patients. For BTKAs, the overall in-hospital complication rate was 1.21% in Medicare/Medicaid patients compared with 0.85% in patients with private insurance. For both UTKAs and BTKAs, the in-hospital complication rate was lower among patients with private insurance compared with Medicare/Medicaid throughout the study period (Figure 3, A and B). After adjusting for covariates including comorbidities, Medicare/Medicaid patients undergoing UTKA had higher odds of in-hospital complications compared with patients with private insurance over the study period (aOR, 1.06, 95% confidence interval, 1.002 to 1.12, *P* = 0.04) (Figure 4). However, for BTKA, the association between insurance type and in-hospital complications was not statistically significant in the adjusted model (aOR, 1.02, 95% confidence interval, 0.79 to 1.3, *P* > 0.05), although complication rates were consistently higher among BTKA patients with Medicare/Medicaid compared with those with private insurance throughout the study period (Figure 3, A).

### Role of Patient Race

**Figure 5:**
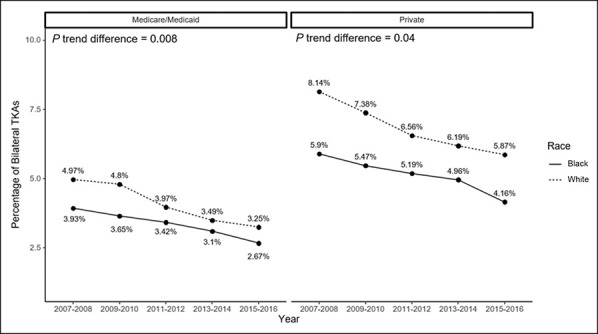
Graph showing bilateral total knee arthroplasty (TKA) trends stratified by primary insurance and race.

We used an interaction model to assess whether the type of insurance differentially affects AAs and Whites and found that race affects utilization differently in patients with Medicaid/Medicare versus private insurance, after adjusting for years; patient age, sex, community median household income, comorbidity; and hospital volume, bed size, region, and teaching/location status. Over the study period, there was higher BTKA utilization among White patients in both Medicare/Medicaid (5.0% in Whites versus 4.0% in AAs, 2007 to 2008; 3.3% in Whites versus 2.7% in AAs, 2015 to 2016) and private insurance (8.1% in Whites versus 5.9% in AAs, 2007 to 2008; 5.9% in Whites versus 4.2% in AAs, 2015 to 2016) (Figure 5). The trend difference between the racial categories is statistically significant in both insurance groups (*P* < 0.05).

The race and insurance interaction was not statistically significant in our stratified models for UTKA and BTKA postoperative complications, after adjusting for covariates. Overall, there is no evidence that race affects BTKA in-hospital complication rates differently by insurance type (*P* > 0.05).

## Discussion

In this national sample spanning 10 years of patients who underwent TKA, we found that although BTKA utilization trends are decreasing overall, they were consistently lower among patients with Medicare/Medicaid insurance compared with those with private insurance. Furthermore, there was a significant race and insurance interaction effect in that White patients with either type of insurance had higher BTKA utilization rates compared with AAs. Although BTKA in-hospital complication rates were higher among Medicare/Medicaid patients than those with private insurance, this relationship was not statistically significant.

To our knowledge, there have only been a few studies that examine the relationship between insurance status and BTKA utilization. Ten years after Medicare decreased the reimbursement for a second arthroplasty performed within 90 days of a first total joint arthroplasty, Della Valle et al^14^ published an initial analysis of BTKA utilization. That study compared the number of BTKA performed in New York State during the 4-year period before and after the policy change. Their analysis, which was limited to Medicare beneficiaries, did not show a difference in utilization rates over time. They concluded that despite a theoretical financial disincentive for surgeons, there were no changes in practices after the reimbursement decrease.

Our study addresses some of the limitations of the Della Valle study by bringing their analysis into the period of Medicare reimbursement decline and expanding the investigation to include patients with private insurance. We report a decline in the proportional use of BTKA in the Medicare/Medicaid population both relative to the Della Valle study and over time (4.8% in 1988 to 6.2% in 1994 versus 4.6% in 2007 to 2008 to 3.1% in 2015 to 2016).^14^ Importantly, by considering the type of insurance, we are able to demonstrate that although the proportion of BTKA among all TKAs declined over the 10-year study, there was an overall lower bilateral utilization rate among patients with federally based insurance.

The effect of insurance status on BTKA utilization during a period of Medicare reimbursement decline was also assessed by Glait et al.^20^ Their analysis of simultaneous THA in New York State from 1990 to 2010 found that the proportional number of cases decreased over time (2.28% in 1990 to 1.51% in 2010). In addition, they found that a higher proportion of patients who underwent simultaneous THA had private versus federally based insurance (66.6% versus 33.4%). This was significantly different than the insurance distribution for unilateral THA, as 53.1% of unilateral THA patients had federal insurance. This percentage increased over time with private insurance covering 72.1% of simultaneous THA cases from 2000 to 2010. While this study found a significant difference in the insurance distribution between simultaneous and unilateral THA cases, a multivariate analysis to determine whether insurance status was individually associated with simultaneous arthroplasty utilization was not conducted. Our study similarly demonstrates a higher percentage of private insurance use among BTKA patients. In addition, after controlling for individual, ecological, and hospital-level variables, our adjusted model found that insurance status is significantly associated with BTKA utilization.

This study is unique in demonstrating the association between BTKA utilization and insurance type. This is an important contribution as it highlights a disparity in care that may increase over time if reimbursement rates change. Although Gait et al demonstrated that the disparity exists, they did not adjust for any confounders in their analysis. Prior literature has indicated that given the concerns for higher complication rates for BTKA, younger and healthier patients are more often selected.^4^ For example, in our cohort, patients with private insurance were more likely to have fewer comorbidities and be admitted to high-volume hospitals. These variables have been documented as important considerations when deciding whether to offer BTKA, as it is generally considered a higher risk surgery compared with UTKA.^9^ Therefore, if privately insured patients are generally healthier than federally insured patients, this may be a reason why they have higher rates of BTKA utilization. However, our analysis is rigorous, and after adjusting for individual-, ecological-, and hospital-level variables, insurance was still associated with differential BTKA utilization in our cohort. Although there is a documented proportional decline in BTKA use, it remains a common procedure. Our study adds insurance status to the list of variables that may affect the ability of patients to access this treatment option.

Our analysis showed lower rates of in-hospital complication rates in private insurance patients compared with Medicare/Medicaid patients throughout the study period for both UTKA and BTKA. This finding is in line with previous literature that has documented higher complication rates in patients with federally funded insurance compared with private insurance.^21,22^ These studies suggest that other factors, such as smoking status, that are not often available in administrative data sets such as NIS, may contribute to the differences in outcomes and complications between insurance types. In addition, despite low overall complication rates, our analysis demonstrated higher complication rates among patients undergoing BTKA compared with UTKA. A similar pattern has been documented extensively in the literature.^9-11^

There was higher utilization of BTKA among White compared with AA patients in both Medicare/Medicaid and private insurance groups over the study period. Various studies have documented decreased utilization of TKA in AAs relative to Whites and noted the higher rate of private insurance in White patients.^23,24^ In addition, as reported by Zhang et al, once insurance was controlled for in the model, race was independently associated with TKA utilization. Our article adds to this literature by analyzing BTKA utilization while considering both insurance and race. Our data would suggest that although insurance status is strongly associated with BTKA utilization, the experience of patients of different races within each insurance group is not the same.

Our study has several limitations. First, NIS includes only procedures in hospitalized patients; therefore, it does not cover same day discharges. However, most TKA patients are admitted to the hospital. Second, NIS does not capture patient-reported outcomes, labs, medications, postdischarge complications, and other surgical factors (e.g., surgical time, surgeon volume, and blood loss), all of which may influence outcomes, and we are unable to comment on these factors. Third, although procedure codes for TKA have a sensitivity of 89% and a specificity of 98%,^25^ complication diagnoses may be misclassified because NIS uses billing information and discharge diagnoses.

In summary, in this national sample spanning 10 years of patients who underwent TKA, we found that although overall BTKA utilization trends are decreasing, they were consistently lower among patients with Medicare/Medicaid insurance compared with those with private insurance. Furthermore, there was a significant race and insurance interaction effect—White patients with either type of insurance had higher BTKA utilization rates compared with AAs. Although BTKA in-hospital complication rates for Medicare/Medicaid patients were higher than for patients with private insurance, this relationship was not statistically significant. This may grow in importance as more patients require TKA and as CMS programs expand national enrollment.

## Supplementary Material

SUPPLEMENTARY MATERIAL
