# Supplementary material for: Medicare/Medicaid Insurance Status Is Associated With Reduced Lower Bilateral Knee Arthroplasty Utilization and Higher Complication Rates
Source: J Am Acad Orthop Surg Glob Res Rev. 2022 Apr 26;6(4):e21.00016. doi: 10.5435/JAAOSGlobal-D-21-00016 (PMC10566829; doi:10.5435/JAAOSGlobal-D-21-00016)
Supplement: SUPPLEMENTARY MATERIAL [file jagrr-6-e21.00016-s001.docx]

Appendix 1. In-hospital complications codes used

| **Complications** | **ICD-9 codes** | **ICD-10-CM codes** |
| --- | --- | --- |
| Acute myocardial infarction | 410 | I21, I22 |
| Venous Thromboembolism | 45340, 45341, 4536, 45342, 45384, 45381, 45386, 45389, 45382, 4539, 45385, 4511, 4512, 4518, 4519 | I801, I802, I803, I809, I822, I823, I828, I829, I824 |
| Wound complications | 99859, 9866, 99667 99830, 99831, 99832, 99833, 99851, 99883 | K6811, T814XXA |
| Device complications | 99666, 9964 | T8481XA, T84115D, T84020A, T84114A, T84218A, T8484XA, T84498A, T84199A, T84011A, T84021A, T8489XA, T8451XA, T84010A, T84091A, T84031A, T84328A, T84030A, T84099A, T84090A, T84428A, T84038A, T84061A, T84050A, T8486XA, T8452XA, T8483XA |
